# Freeze Substitution Accelerated via Agitation: New Prospects for Ultrastructural Studies of Lichen Symbionts and Their Extracellular Matrix

**DOI:** 10.3390/plants12234039

**Published:** 2023-11-30

**Authors:** Siegfried Reipert, Daniela Gruber, Norbert Cyran, Brigitte Schmidt, Rosa de la Torre Noetzel, Leopoldo G. Sancho, Michal Goga, Martin Bačkor, Katy Schmidt

**Affiliations:** 1 Cell Imaging and Ultrastructural Research, University of Vienna, A-1030 Vienna, Austria; daniela.gruber@univie.ac.at (D.G.); norbert.cyran@univie.ac.at (N.C.); brigitte.schmidt@univie.ac.at (B.S.);; 2 Department of Earth Observation, National Institute for Aerospace Technology, 28850 Madrid, Spain; torrenr@inta.es; 3 Section of Botany, Faculty of Pharmacy, University Complutense Madrid, 28040 Madrid, Spain; sancholg@farm.ucm.es; 4 Institute of Biology and Ecology, Pavol Jozef Šafárik University, 040 01 Košice, Slovakia; michal.goga@upjs.sk (M.G.); martin.backor@uniag.sk (M.B.); 5 Institute of Biotechnology, Faculty of Biotechnology and Food Sciences, Slovak University of Agriculture, 949 76 Nitra, Slovakia

**Keywords:** lichens, algal photobionts, extracellular matrix, freeze substitution, sample preparation, transmission electron microscopy

## Abstract

(1) Background: Lichens, as an important part of the terrestrial ecosystem, attract the attention of various research disciplines. To elucidate their ultrastructure, transmission electron microscopy of resin-embedded samples is indispensable. Since most observations of lichen samples are generated via chemical fixation and processing at room temperature, they lack the rapid immobilization of live processes and are prone to preparation artefacts. To improve their preservation, cryoprocessing was tested in the past, but never widely implemented, not least because of an extremely lengthy protocol. (2) Methods: Here, we introduce an accelerated automated freeze substitution protocol with continuous agitation. Using the example of three lichen species, we demonstrate the preservation of the native state of algal photobionts and mycobionts in association with their extracellular matrix. (3) Results: We bring to attention the extent and the structural variability of the hyphae, the extracellular matrix and numerous crystallized metabolites. Our findings will encourage studies on transformation processes related to the compartmentation of lichen thalli. They include cryopreserved aspects of algal photobionts and observations of putative physiological relevance, such as the arrangement of numerous mitochondria within chloroplast pockets. (4) Conclusions: In summary, we present accelerated freeze substitution as a very useful tool for systematic studies of lichen ultrastructures.

## 1. Introduction

Insights into the morphology and ultrastructure of lichens using transmission electron microscopy (TEM) are crucial for understanding the symbiotic interactions between photobionts (algae or cyanobacteria) and fungi (mycobionts). Besides the elucidation of functional aspects of the symbiosis itself, TEM could also be used in combination with PCR techniques to elucidate taxonomy, contributing, for instance, to a delimitation of the diversity of algal photobionts which coexist in the same thallus [1]. Previously, it has been suggested that the application of genetic markers in combination with ultrastructural techniques such as TEM should be standardized [2]. Cleary, such a demand poses a challenge for TEM preparation, concerning both the choice of basic preparation concepts and the necessity of adaptation for each variant of over 18.000 lichen species worldwide [3].

The preparatory foundations for the investigation of lichen ultrastructures via TEM were laid through the pioneering work of lichenologists and electron microscopists in the late 1960s, 1970s and 1980s (for review: [4]). Based on techniques relying on chemical fixation and resin embedding, the variability of lichen ultrastructures in relation to experimental, environmental and seasonal conditions was explored in the past [5]. Notably, studies of TEM sections of embedded thalli provided insights into the fungal–photobiont interaction beyond electron microscopy (EM) surface imaging techniques [6]. In the 1990s and the beginning of the 21st century, TEM became part an increasingly multimodal imaging approach for lichens encompassing various TEM, SEM and light microscopic techniques (for review: [7]).

Until now, the overwhelming majority of TEM data originate from lichen samples processed at ambient temperatures. Conventional chemical fixation and embedding protocols were adapted for a wide variety of lichen species, such as *Parmelia sulcata* [6]; *Ramalina farinacea* [8]; *Pseudevernia furfuracea* [9]; *Usnea longissima* and *Ramalina menziesi* [10]; *Parmotrema pseudotinctorum* [11,12]; *Cladonia convoluta-C. foliacea* [13]; *Gyalectidium- paolae* [14]; *Ramalina fraxinea* [2]; *Xanthoria parietina* [15,16,17]; *Xanthoria elegans* [18]; *Sticta canariensis*, *Leptogium cyanescens* and *Endocarpon pusillum* [19]; *Circinaria gyrosa* [20]; and *Lobaria pulmonaria* [21]. All of these publications share a common use of aldehydes as primary fixatives, sometimes alongside prolonged dehydration and infiltration, used frequently with low-viscosity resin to deal with the constraints of algal and fungal cell walls. As a consequence, the samples not only lack the instant immobilization of the living state; they also display shrinkage and the release of biological material of various degrees, as well as a loss of the turgescent state of the photobionts.

Since cryopreparation is superior to chemical processing at ambient temperatures with regard to the preservation of most biological samples [22,23,24], the usefulness of rapid cryoimmobilization and low-temperature dehydration and fixation for studies of lichens’ ultrastructures should be thoroughly evaluated. In 1994, Honegger and Peter chose *Lobaria virens* with its photobiont, *Dictyochloropsis reticulata*, as a particularly demanding test organism for comparative conventional and cryopreparation [25]. While the cell organelles of the mycobiont and photobiont shrank during conventional preparation, they retained their turgescent state and tight outlines after cryopreparation. Despite these advantages, the protocol was not practicable for wide-spread application, since the freeze substitution (FS) of the frozen lichen samples in methanol containing 4% acrolein at −85 °C lasted at least three weeks, and was followed by a gradual warming and low-temperature fixation in methanol containing 2% acrolein and 2% osmium tetroxide at −50 °C for at least 24 h. Slightly modified FS protocols apply even longer substitution (40–116 days in a −85 °C freezer) and warm-up times (47–100 h) to plunge-frozen foliose macro-lichens from the Lower Devonian period [26,27]. In 2002, de los Ríos and Ascaso tested significantly shorter protocols by using either pure acetone or methanol with 2% osmium tetroxide and 0.1% uranyl acetate as the substitution medium [4]. The substitution times of 72 h and 20 h at −90 °C, respectively, were in the range of FS protocols commonly applied to other cell and tissue specimens at the time. The outcome, represented by a single micrograph of *Trebouxia* algae from *Xanthoparmelia porkonyi*, however, did not promise significant preparative improvement.

While the pioneering work mentioned above did not initiate a breakthrough of using cryopreparation for entire lichens, cryofixation and FS were applied to isolated algal photobionts. The ultrastructure of *Trebouxia aggregata*, an algal genus most commonly present in lichens worldwide [28], has been elucidated by Sluiman and Lokhorst, who applied FS in OsO_4_/acetone for 2–3 days to these plunge-frozen algae [29]. More recently, the microalgae *Trebouxia lynnae* (formerly *Trebouxia* sp. TR9) [30] and *Coccomyxa simplex,* isolated from the lichens *Ramalina farinacea* and *Solorina saccata*, respectively, were high-pressure frozen and freeze-substituted with 1.5% OsO_4_ in acetone for 54 h [31]. High-pressure freezing (HPF) and FS for 68 h were also applied to the protists *Saccharomycomorpha psychra* n. g., n. sp., isolated from the Arctic lichens *Cladonia pocillum* [32].

Lately, the utilization of cryopreservation for ultrastructures has gained momentum by accelerating the FS process. McDonald and Webb shortened the FS of selected tissues to three hours or even less via sample agitation in a dry-ice-filled bucket on a laboratory shaker [33]. A few years later, Goldammer et al. performed continuous sample agitation within the cryochamber of an automated freeze substitution system (AFS) [34]. Along with other samples, they included cultured algae and those living in symbiosis with the unicellular ciliate *Paramecium* in the prototype testing of an agitation module inserted in the AFS [34,35]. Similar to the experimental setup by McDonald and Webb [33], the FS of algae and yeast under agitation became a matter of hours rather than days. Moreover, agitation in an AFS offered flexibility in the prolongation of the substitution process as required for samples with strong barriers to media exchange, such as the eggshells of brine shrimp embryos of *Artemia franciscana* and the cell walls of anthers of *Arabidopsis thaliana* [35].

Based on the experience that sample agitation accelerates the FS of fungi and algae [34,35], we hypothesized that processing photo- and mycobionts in their symbiotic relation could also be performed more rapidly, free of ice crystal damage and with similar positive effects on structural preservation. As a result of the application to lichens, we expected less shrinkage, as well as reduced wash-out and aggregation of the biological material compared to conventional processing at room temperature. Moreover, the turgidity of cells and cell organelles should be maintained, as this is the case when using other cryo-techniques, such as low-temperature scanning electron microscopy (LTSEM), which was recently applied to microalgae isolated from lichens [36], and quick-freeze deep-etch electron microscopy (QFDEEM) [37]. The latter revealed the extended, diverse nature of lichens’ extracellular matrix (ECM) that is thought to function in complex processes of signalling and recognition (for review: [38,39]). Since FS favours the preservation of biopolymers and sugars, e.g., demonstrated for algal starch [34], we also expected significantly improved conservation of the thallus-forming, biopolymeric structures in lichens. In short, we wanted to test the hypothesis of whether more informative results could be achieved in a shorter timeframe to improve lichen research.

Here, we present a valid protocol for FS under agitation as a proven and tested starting point to become both an inspiration for ultrastructural research and a connecting link to other EM cryo-techniques involved in the exploration of lichens. To demonstrate its potential, we chose lichen species differing in their habitats that are already the subject of cutting-edge space research (*C. gyrosa* [20]) and environmental research (*X. parietina* [16]; *U. antarctica* [40]). Morphological and ultrastructural features, possibly related to non-quantified differences in the hydration status (hydrated *X. parietina*; partially rehydrated *C. gyrosa* and *U. antarctica*) point to future applications that could explore ultrastructural implications of poikilohydrous water relations in lichens adequately, as it was predicted by Honegger and Peter [25] almost three decades ago.

## 2. Results

We cryopreserved lichens using a double-directed approach: (i) we demonstrated how FS could preserve fungi and photobionts inclusively of the ECM “glue”; (ii) we retained a high level of integrity of the sections, enabling studies of cryopreserved lichens. To achieve this, we adjusted the temperature/time schedule of the FS process through sample agitation in an AFS, and we employed an oscillating diamond knife [41] to reduce tensions causing disruption during the sectioning of the epoxy resin-embedded lichen thalli that had previously been identified as a major technical problem [38].

### 2.1. FS under Continuous Agitation—A Means to Cryopreserve Lichens for TEM

Initial tests of the conditions for freezing and FS were performed with *X. parietina*, a foliose lichen that is widely distributed and readily available. Based on experience with the FS of demanding plant and animal samples, we applied FS under agitation overnight instead of a very short program that suits single cells, such as algae [34,35]. Despite the prolongation of the substitution time to 10 h (Table 1), the sections displayed severe tearing of the cells that could not be linked to an insufficient infiltration of the cells with resin. Partially intact cells, however, indicated that the ultrastructure was well-preserved and free of freezing- and FS-related artefacts. (Figure S1). Aiming for a protocol that was suitable for systematic studies but remained within a practicable timeframe, we extended the FS process to 60 h (Table 1 and temperature/time schedule, Figure S2). This measure aimed at the withdrawal of residual water molecules that were supposed to bind more strongly to certain lichen structures and, consequently, to result in a more uniform hardening of the epoxy resin throughout the samples. As a result, sections for light microscopy (2.5 µm in thickness) could be taken with a glass knife more easily. If the section size was kept small (less than 0.5 mm wide), the damage during thin and ultrathin sectioning could be reduced but not completely excluded.

### 2.2. Improvement in Section Quality by Using an Ultrasonic Diamond Knife

As a result of sectioning with an ultramicrotome, otherwise well-preserved samples frequently displayed tears at the interfaces between the cell walls and the cytoplasm of the algae and fungi, as well as in the ECM. Rips were also abundant in hyphae at the interfaces between the membranes of large internal vacuoles and their electron-lucent homogeneous content, and inside algal microbodies. The issue was resolved by using an oscillating diamond knife for sectioning at a self-adjusted resonance frequency between 23 and 27 kHz and a freely selectable ultrasound amplitude [41]. Although the amplitude could be raised up to the maximum with a setting of 30 V, we achieved the best results with a moderate voltage of 6 V, as recommended for epoxy resin sections (personal communication, Helmut Gnaegi). Higher amplitudes (tested for 12 and 24 V) led to disturbing periodic shatter marks on the sections. For studying profiles of thalli, we had to cut sections of approx. 1 mm width, which is twice the recommended size. Distortions were still reduced to a level that enabled meaningful ultrastructural studies. Intracellular damage of the algal microbodies and vacuoles of hyphae could be prevented almost completely.

### 2.3. Cryoimmobilized Hyphal/Algal Junction in X. parietina Displays Patterns of Intraparietal Cell Wall Contact

**Figure 1:**
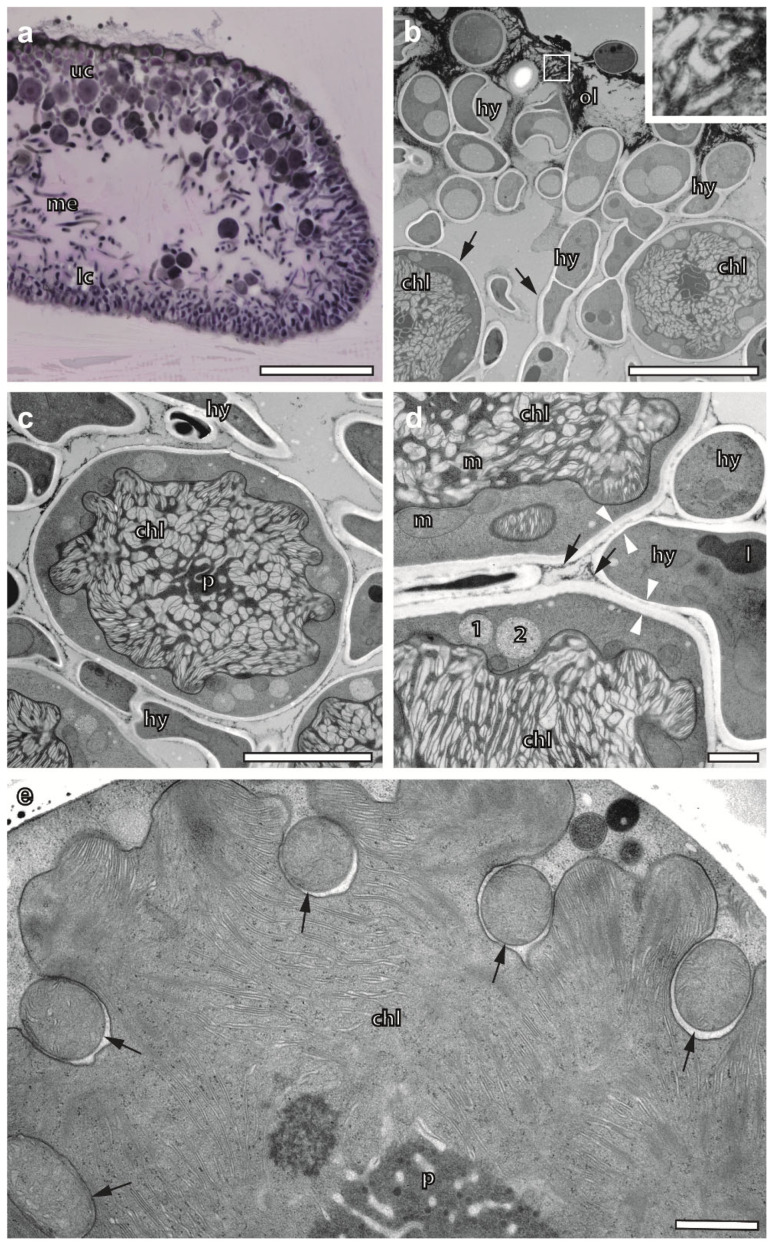
High-pressure frozen *X. parietina*, freeze-substituted and embedded in epoxy resin. (**a**) Light microscopic section of a pseudo-meristem stained with toluidine blue consisting of upper cortex, medulla with loosely arranged fungal hyphae and lower cortex. Scale bar, 100 µm. (**b**–**e**) Transmission electron microscopy of ultrathin sections. (**b**) *Trebouxia* sp. with prominent chloroplasts and hyphae of the upper cortex located next to the outer layer. The hyphae are rich in vacuoles and osmophilic droplets. Both algae and fungi are lined with bright, electron-lucent cell walls. Arrows point to thin layers of electron-dense substance decorating the cell walls of both photobionts and mycobionts. The outer layer contains crystal-like inclusions. Insert: Crystal-like inclusion of the boxed area shown in detail. Scale bar, 10 µm. (**c**) *Trebouxia* photobiont encircled by hyphae. Some of them are in contact or fused with their cell walls. Scale bar, 5 µm. (**d**) Algal/fungal contact in detail. Note a thin electron-dense layer of bio-minerals lining the cell walls of algae and fungi that is thinner or even interrupted at cell/cell contact sites, so-called junctions. White arrows mark those junctions that seem to be progressed in fusion; the fungal cell wall is locally thinned. (**e**) Lobed chloroplast with densely stacked thylakoid membranes and pockets filled with one mitochondrion each marked with arrows. ol—outer layer; uc—upper cortex; me—medulla; lc—lower cortex; hy—hypha; chl—chloroplast; p—pyrenoid; m—mitochondrion; 1, 2—microbodies; l—lipid droplet. Scale bars in d and e, 1 µm.

Employing FS under agitation over the weekend, we obtained intact sections of *X. parietina* for both EM and (light microscopy) LM, as exemplified using a light microscopic overview of a pseudo-meristem (Figure 1a). As a consequence of cryopreparation, organelles, such as vacuoles in the hyphae (Figure 1b) and mitochondria and microbodies in the algae (Figure 1d,e), displayed a typical turgid appearance without wrinkles in their lipid membranes. Since the vast majority of algae and hyphae were structurally not impaired in the resin sections, their interaction could be evaluated within the whole cross-section via TEM. Details of the algae and hyphae underneath the cortex covered by biofilms with crystal-like inclusions are shown in Figure 1b. Multiple fusion sites between the round and elliptically shaped profiles of hyphae and the cell walls of algae, also called junctions [42], can be recognized in Figure 1b,c. Interestingly, none of the many contact sites of the cell walls suggested the existence of vesicular transport processes. The hyphal profiles indicated tangential cell wall contact rather than a frontal approach, or even the orthogonal entering of the algal cell wall. A thin layer of an electron-dense substance decorating the outside of the cell walls was observed, allowing us to distinguish if fusion between the hyphae and algal cell walls was established or not. It resembled similar observations of freeze-etched lichens interpreted as hydrophobins [43]. Another criterion for fusion could be the exclusive reduction in thickness of the fungal cell wall at putative fusion sites in *X. parietina* (Figure 1d). In summary, the mycobiont/photobiont contact in *X. parietina* met the patterns of intraparietal cell wall contact as described previously [44,45].

### 2.4. Cryopreparation Suits the Preservation of Photobionts at Various Degrees of Hydration

The biological activity of most of the *Trebouxia* sp. in *X. parietina* was indicated based on their turgescent overall appearance; large chloroplasts with a lobed shape; pyrenoids and thylakoid organization at various, yet unexplored, stages; mitochondria; and many microbodies with granular content (Figure 1). Frequently, mitochondria could be found in pockets formed by the lobed chloroplast. The membranes of both organelles displayed close proximity over the mitochondrial perimeter, with the exception of narrow openings towards the algal cytoplasm (Figure 1e). This observation coincided with densely packed thylakoid membrane stacks and well-developed, mostly globular pyrenoids.

In addition to fully hydrated *X. parietina*, two lichens (*U. antarctica* and *C. gyrosa*), rehydrated to an unknown extent after long-term dry storage, were chosen for further testing. In particular, the morphology and ultrastructure of the algal photobionts of *U. antarctica* differed significantly from observations of *X. parietina*. While some algae indicated a slow recovery from the dried state, others showed signs of irreversible damage that might be caused by long-term storage.

**Figure 2:**
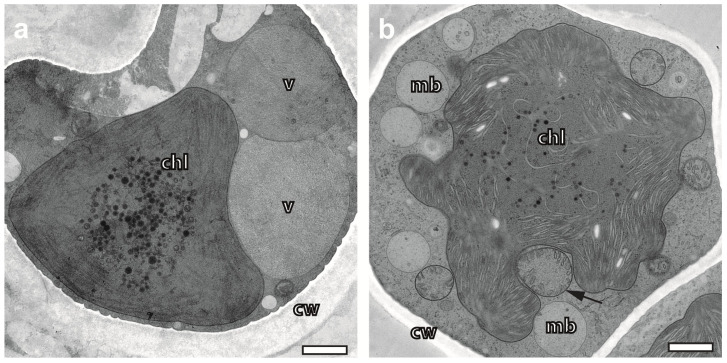
Rehydration-dependent morphological and ultrastructural variety of photobionts in cryoimmobilized, freeze-substituted lichens. (**a**) Alga of *U. antarctica* showing hallmarks of drying. The whole cell is kidney-shaped and endowed with an electron-dense chloroplastwith a completely smooth, rounded shape. Mitochondria are missing in the plane of the section. Two large vacuoles are filled with granular content. (**b**) Alga in rehydrated *C. gyrosa* displaying a lobed chloroplast with densely packed thylakoid membranes. The dispersed pyrenoid in its centre contains only a small amount of electron-dense pyrenoglobuli. An arrow points towards a mitochondrion within a pocket formed by the chloroplast. Chl—chloroplast; mb—microbody; cw—cell wall; v—vacuole. Scale bars, 1 µm.

In *U. antarctica*, we observed kidney-shaped, electron-dense algae (Figure 2a and Figure S3b) similarly to isolated and desiccated cultures of *Trebouxia* sp. that were cryo-processed [31]. The alga in Figure 2a appears to be at the start of rehydration. It contains giant vacuoles preserved via cryo-preparation, but it remains uncertain whether its vitality may be fully restored under the given circumstances. Other photobionts with some morphological similarities displayed a rounded overall shape (Figure S3).

The algal photobiont in *C. gyrosa* (Figure 2b) seemed to be advanced in rehydration, since it displayed a more turgescent overall morphology and a lobed chloroplast with a mitochondrion located within its pocket. The pocketing of the mitochondrion by the chloroplast indicates an arrangement that is otherwise typical for *Trebouxia* sp. in a state of hydration (Figure 1e and Figure 6c). The photobionts of *U. antarctica*, in contrast, contained rounded or even globular chloroplasts without any lobes, mitochondria with disordered cristae, some individual very large vacuoles and microbodies. Besides that, many algae contained small vesicles aligned along the cell membrane (Figure 2a and Figure S3).

**Figure 3:**
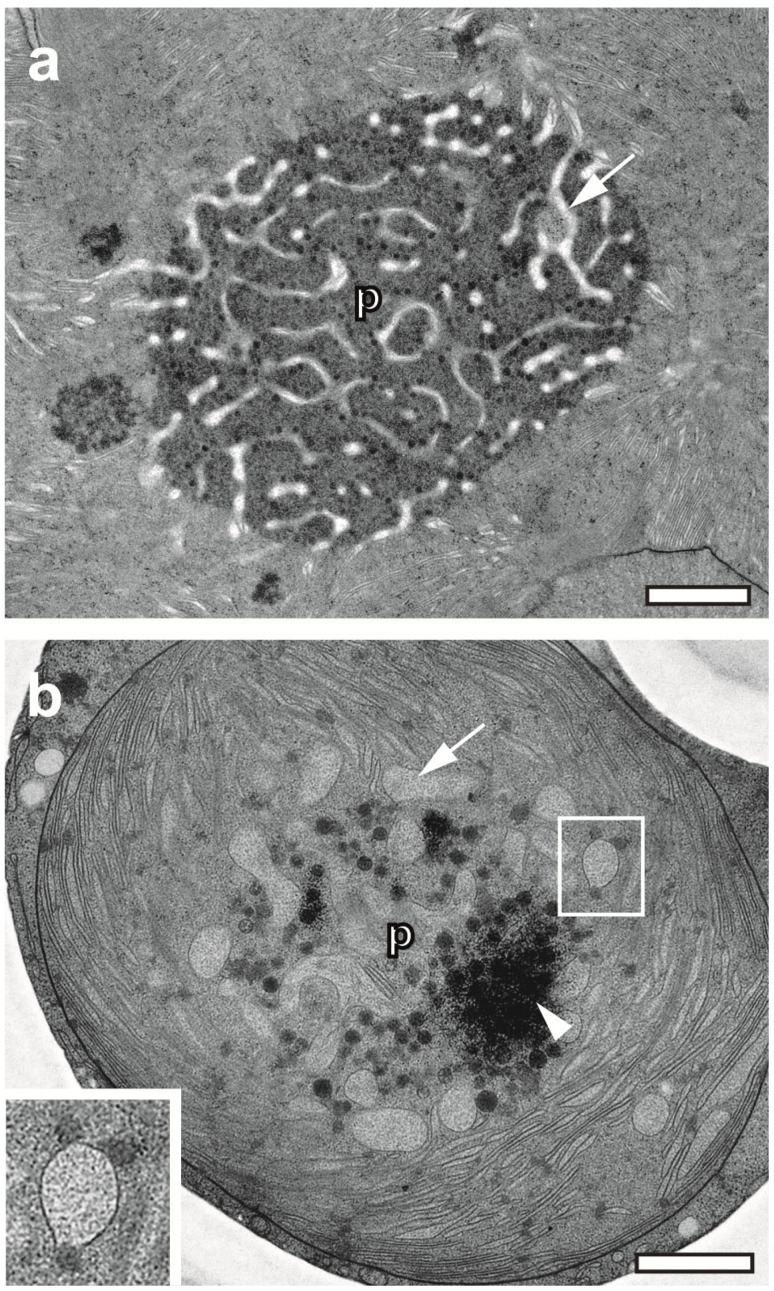
Cryopreservation of unexplored transformation of processes of pyrenoids in the context of photobiont physiology. (**a**) Gigantea-type pyrenoid [46] of *Trebouxia* sp. in hydrated *X. parietina* interspersed with membranes and numerous electron-dense pyrenoglobuli. (**b**) Pyrenoid of a partially dehydrated algal photobiont in *U. antarctica* with typical kidney-shaped overall morphology, as displayed in full in Figure S3. Numerous electron-dense pyrenoglobuli are clustered (arrowhead), while other seem to fuse with blistered membranes (see insert), perhaps as part of a transformation process of the pyrenoid. p—pyrenoid. Scale bars, 1 µm.

Moreover, the status of hydration might influence pyrenoid organization. While the hydrated algae of *X. parietina* show compacted globular pyrenoids interspersed with membranes and numerous electron-dense pyrenoglobuli (Figure 3a), the rehydrated algae of *U. antarctica* displayed a blistering of the thylakoid membranes in association with clusters of electron-dense pyrenoglobuli (Figure 3b). As indicated in the insert (Figure 3b), some pyrenoglobuli appeared to be fused with the membranes. Should this observation represent a putative transformation process, it points to a key feature of cryopreparation, namely the instantaneous immobilisation of fast living processes.

Taken together, our data on photobionts of various lichen species demonstrate that the FS protocol works for lichen samples at various degrees of hydration. Future experiments exploring cryoimmobilized ultrastructural processes related to desiccation and stress are therefore feasible.

### 2.5. FS Enables Superb Preservation of the ECM in Association with Hyphae

**Figure 4:**
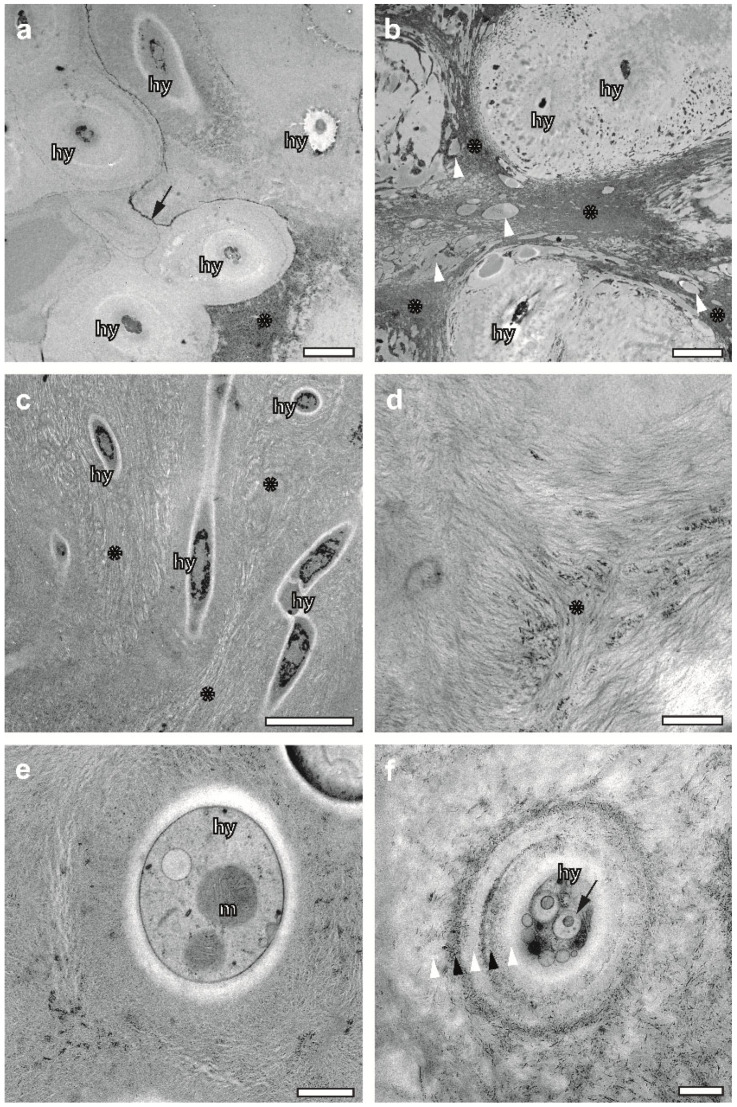
Variability of both hyphae and ECM structures of lichens preserved via accelerated freeze substitution. (**a**) ECM domain of incompletely rehydrated *U. antarctica* with “acellular” hyphae embedded in a gelatinous ground substance. Fascicle-like wrappings of hyphae are separated by electron-dense layers (arrow) of biominerals that vary in thickness. Asterisk—biomineral layer oriented in parallel to the section plane. Scale bar, 2 µm. (**b**) ECM domain within the same sample as in A, characterized by strongly conglutinated material between hyphae that are seemingly progressed in transformation towards “acellular” structures. Arrowheads mark inclusion within the conglutinated ECM material. Scale bar, 2 µm. (**c**–**f**) *X. parietina*. (**c**) Hyphae filled with electron-dense content of unknown origin within an ECM continuum of partially agglutinated fibres (asterisk). Scale bar, 5 µm. (**d**) Densely woven ECM network made of individual fibres converging in a centre (asterisk) that is rich in electron-dense bio-minerals. (**e**) Cross-sectioned hypha endowed with a clearly visible cell membrane and diffusely outlined electron-lucent cell wall that includes a cytoplasm filled with organelles such as mitochondria, vacuoles and ribosomes. (**f**) Hypha wrapped in multi-layered material varying in its electron density (white arrows and black arrowheads). Note a very disperse outer wall layer and an innermost electron-lucent layer that resembles the cell wall in e. A cell membrane is missing. The cytoplasm contains small vesicles and autolytic vacuoles with vesicular content. hy—hypha, m—mitochondrion. Scale bars in (**d**–**f**), 500 nm.

FS proved to be a means for the preservation of the ECM at large in association with the outer cell wall layers of hyphae. This included both the gelatine-like domains separated by interphases with inclusions of electron-dense metabolites (Figure 4a), and large areas of more or less strongly conglutinated fibrous domains of the medulla (Figure 4b,c), seemingly embedded in a ground substance. While chemical fixation and processing at room temperature bears the risk of aggregation of fibrous structures and filaments, FS under agitation is able to preserve filamentous structures without artefactual aggregation. As demonstrated in Figure 4d, individual fibres, not seen before in conventional sample preparation, are clearly resolved in the TEM at higher magnification.

**Figure 5:**
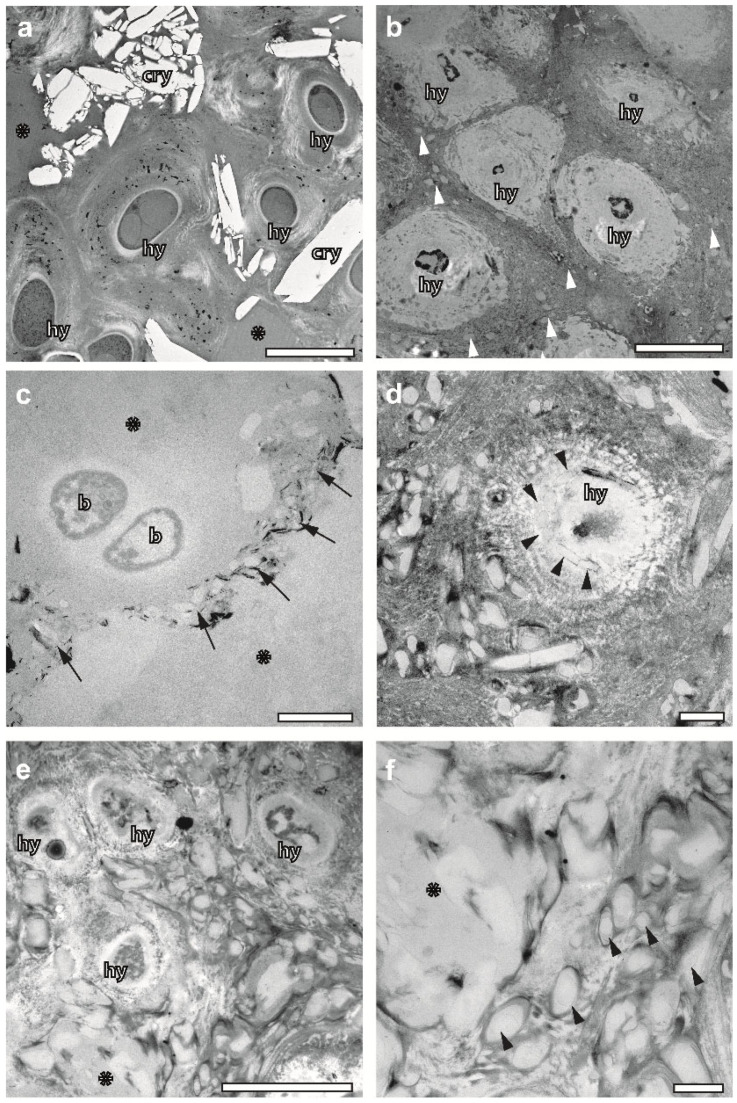
Preservation of crystal-like inclusions inside thalli of cryoimmobilized, freeze-substituted lichens. (**a**) Large electron-lucent Ca-oxalate crystals embedded in the ECM of *C. gyrosa*. Note also numerous small, electron-dense entities of unknown origin and cross-sectioned hyphae with large vacuoles inside. Gelatinous ECM domains are marked with asterisks. Scale bar, 5 µm. (**b**–**f**) Crystal-like entities of unknown origin in the medulla of *U. antarctica:* (**b**) Conglutinated fibres and radial wrapping of hyphae by ECM material coalesced as a continuum. In between, there are many small, rhomboid-like entities located (arrowheads). Scale bar, 5 µm. (**c**) Gelatinous ECM (asterisks) within the same sample as in b, interspersed with channel-like interfaces that are filled with small, electron-lucent rhomboids and black, needle-like crystals. Scale bar, 1 µm. (**d**) Hypha with rhomboid crystals inside (arrowheads) and next to it. Scale bar, 1 µm. (**e**) Accumulation of numerous crystal-like inclusions within conglutinated ECM wrapping hyphae. Asterisk marking a “gelatinous” area that is displayed in more detail in f. Scale bar, 5 µm. (**f**) Note faint linings of crystal-like entities within gelatinous “tears” (arrowheads) and modulations in contrast within the “gelatinous” domain (asterisk). cry—crystal; hy—hypha; b—bacterium. Scale bar, 1 µm.

Moreover, our results of the structural diversity of the lichen ECM agree with QFDEEM data with regard to so-called “fog” [45]. According to Goodenough and Roth, the peculiar “fog”-like ground substance is likely to represent a liquid-glass-like mixture of secondary metabolites (synthesized by the fungi), polyols (synthesized by the algae) and polyol-sequestered water. The “fog” seems to be represented in freeze-substituted lichens as a homogenous, gelatine-like substance (Figure 4a and Figure 5c).

### 2.6. FS Draws Attention on Structural Diversity of Hyphae

Conventional sample preparation might preserve the cell walls of hyphae up to a degree that allows for studies of their thickening with respect to thallus development and age [5]. Here, we demonstrate that the protoplasmic content of hyphae can and should be included in developmental studies, since FS preserves its variance throughout the thallus. While hyphae at the upper cortex of *X. parietina* possess a cytoplasm filled with ribosomes, vacuoles, lipid droplets, vesicles, mitochondria, etc. (Figure 1b and Figure 4e), the hyphae of the medulla display electron-dense, irregular entities of unknown origin, and they are free from organelles and ribosomes (Figure 4a–c). As demonstrated in Figure 4f, hyphae undergo a transformation, including autolytic processes in their cytoplasm, the loss of their cell membrane and a layered radial thickening of their cell walls. Accelerated FS offers a possibility for systematic studies of these processes in the context of thallus development. It resembles observations based on QFDEEM, reporting “honeycomb” hyphae, and debris containing “acellular struts” instead of “regular hyphae” [45].

### 2.7. FS Preserves Crystalline Metabolites inside the Thallus

We applied FS to *C. gyrosa,* known as a lichen species containing Ca-oxalate crystals in the medulla, as detected previously using Raman spectroscopy [47]. Whilst the TEM of conventionally embedded samples could not confirm this finding [20], the freeze-substituted samples contained numerous extracellular crystals, large in size and electron-lucent in their appearance (Figure 5a). Subsequent studies of resin-embedded samples via EDX found an enrichment of Ca in these crystals in support of their Ca-oxalate nature (Figure S4).

Studies of *U. antarctica* underlined the suitability of cryopreparation for studies of crystallization in lichens, in more general terms, since we found large amounts of various crystal-like inclusions differing from those in *C. gyrosa* in size, shape and electron density. EDX analyses did not detect Ca as part of these inclusions that could give a hint towards a Ca-oxalate nature (Figure S4). As demonstrated in Figure 5b–e, most of these crystals are lined up extracellularly between the fascicle-like wrappings of neighbouring hyphae. They are smaller and less electron-lucent compared to Ca-oxalate crystals of *C. gyrosa*. Those of the rhomboid type display a very thin, dark lining of their contours, likely caused by contrasted binding proteins (Figure 5c,d). FS also preserved plate-like crystals and very electron-dense metabolites in form of small platelets and needles (Figure 5c). Strikingly, some hyphal profiles in a progressed state of “acellularity” also contained crystal-like inclusions (Figure 5d). They seem to line up radially inside a hypha and its dispersed cell wall, while the surrounding ECM is filled with larger crystals of the same type. While Figure 5d gives the impression that crystals are generated within “acellular” hyphae columns, there is also evidence of the transition processes of crystals outside, in the ECM. Figure 5e shows an area with plenty crystalline entities. As seen in detail (Figure 5f), some of them seem to be immersed in a homogeneous, perhaps gel-like ground substance; putative crystallization frontiers are recognizable within otherwise rounded entities in the form of “tears”. Whether this observation relates to crystal growth or dissolution as a consequence of the rehydration of lichens cannot be concluded from these micrographs.

## 3. Discussion

Sample agitation has been proven as a means to significantly accelerate the FS of biological samples [33,34,35]. Lichens by no means make an exception. Whilst the pioneering protocol applied by Honegger and Peter lasted over a couple of weeks under static conditions [25], the lichens chosen for our demonstration underwent FS under agitation in acetone/OsO_4_ over just one weekend (ca. 60 h). Shorter processing times likely result in severely disrupted resin sections, which only enable studies of fragments or individual cells. The sporadic encounter with undamaged cells within such sections makes their assignment to the thallus architecture difficult. However, measures in favour of section integrity (cutting particular small sections, use of Formvar-coated grids with narrow mesh wide, etc.) may suit the visualization of individual photobionts or hyphae in TEM.

So far, only a tiny fraction of the more than 18.000 lichen species [3] has been studied via TEM. Particularly fragile lichens may already disintegrate during dissecting for HPF. Some lichens may require the addition of crosslinking aldehydes to the substitution medium, or a prolongation of the dehydration and/or resin infiltration. From our point of view, this protocol should be appropriate for various lichen species, including those endowed with a particularly strong extracortical layer and an acellular inner stem, such as *U. antarctica*. In any case, however, FS under agitation is a promising concept to start with.

We agree with the explanation given by Spribille et al. that disruption during the sectioning of chemically fixed epoxy resin-embedded lichens is caused by the differential hardness of the ECM and the cell walls of fungi [38]. Apparently, these differences cannot be compensated for by the hardness of the resin mixture itself. Since humidity has an overriding softening effect on the polymerization of epoxy resin, we wonder whether unwanted residual water molecules are the cause for local variations in sample hardness. Given the extraordinary dynamic and structural variability of water’s interaction with biomolecules, it is reasonable that certain structures, organelles, etc., within cells and tissues bind water more intensely [48]. Consequently, it requires particular efforts for completing dehydration with organic solvents. Hydrophilic, extracellular polymeric substances of the lichen’s ECM with its conglutinate pseudoparenchyma [37,49] are presumably such structures. Either they are washed out and destructed prior to the lichen´s embedding in resin (as happens during conventional processing at ambient temperature) or they pose a challenge for sectioning that can be tackled with extraction-limited low-temperature dehydration during FS in combination with the sectioning technique itself.

For all three lichen species, we performed conventional chemical fixation and embedding at room temperature (Figure S5). Moreover, we compared our FS results with previously published TEM data. The results for *X. parietina* by Vannini et al. indicate severe preparation problems [16]. Cryopreparation would exceed the possibilities for comparative studies with the ozone-treated *X. parietina* samples. Both *C. gyrosa* as an extremophile organism and *X. parietina* were chosen for studies of the limits of terrestrial life in space and Mars-like conditions [18,20]. Cryopreparation demonstrates superiority in the preservation of the ultrastructures of both lichen species. Consequently, it will extend the list of ultrastructural features that can be investigated in future extra-terrestrial studies. *U. antarctica* certainly pose an even greater challenge for sample preparation. Gielwanowska and Olech presented a single TEM micrograph of a strongly degenerated photobiont as the only yield for *U. antarctica* after aldehyde fixation and embedding at room temperature [50]. FS, in contrast, provided information on structurally intact photobionts and hyphae embedded in their ECM surroundings. Moreover, it preserved a diversity of crystalline substances within the ECM via FS.

**Figure 6:**
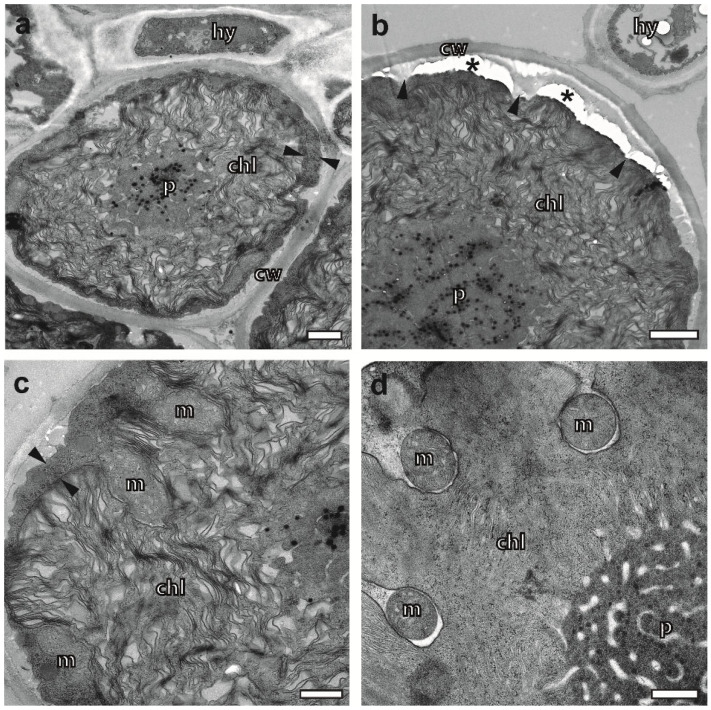
Photobiont ultrastructure of *X. parietina* chemically fixed and embedded at room temperature for comparison with cryopreparation. (**a**) Section of a photobiont alga displaying a large chloroplast surrounded by a narrow band of cytoplasm (between two arrowheads). (**b**) Detail of an algal photobiont with its cytoplasm locally disrupted from the cell wall. Arrowheads point towards “bridges” between the cell wall and the cell membrane of the shrunken cell. Asterisks mark holes in the resin. (**c**) Detail of a chloroplast with wrinkled thylakoid membranes and lumen variability in thickness. Mitochondria inside chloroplast pockets are barely visible and almost merged with the chloroplast membrane. (**d**) Detail of a chloroplast cryopreserved through HPF and FS. Thylakoid membranes are organized in ordered stacks and they confine very thin layers of lumen. Chloroplast pockets contain mitochondria with smooth membranes and clearly visible gaps between their outer membranes and the chloroplast. chl—chloroplast, p—pyrenoid, hy—hypha, m—mitochondrion, cw—cell wall. Scale bars in (**a**,**b**), 1 µm. Scale bars in (**c**,**d**), 500 nm.

The shrinkage of the algal cytoplasm (often coinciding with irregular folding and the aggregation of thylakoid membranes) points to preparation artefacts (Figure 6a–c). Resulting tensions between the rigid cell wall and the cell membrane, indicated by “bridges” (Figure 6b), eventually lead to the disruption of the interface and free space between the cell wall and the cytoplasm. If this scenario is accompanied by lesions of the cell membrane and weak chemical fixation, the accidental release of cytoplasmic material into the free space is plausible. This leads to the question of how to distinguish between physiologically relevant cell secretion and such an artefact. Honegger and Peter demonstrated that shrinkage artefacts can be avoided with FS [25]. Accordingly, we did not observe any electron-lucent gaps between the cell wall and cytoplasm of the algae in the three more rapidly freeze-substituted lichen species (Figures S3 and S6). Therefore, we suggest verifying previous observations of “secretion zones” [11,17] or “secretory space” [1,12,13] by using FS to exclude the possibility that they are the result of structural damage.

A striking improvement that comes with accelerated FS concerns the ECM of lichens. It was demonstrated that the wrapping of individual hyphae by the ECM is interconnected to an extended ECM continuum. Strikingly, both “gelatinous” and filamentous domains of the ECM were preserved. Variance in the thickness of filamentous structures throughout the thalli provided evidence for a process of conglutination. While conglutination is a widely accepted phenomenon [10,27], the usability of conventional chemical sample preparation to study its formation has limits. Conclusively, the data obtained via accelerated FS fit best with so-called microfibrillar gelatinous sheaths in cyanobacterial lichens preserved using replica techniques [27].

Bearing in mind that ECM components are polysaccharides by nature, typically consisting of D-glucose, D-galactose and D-mannose [38], this result is in line with a positive outcome we experienced with the cryopreservation of other structured sugars, such as pyrenoid starch sheaths and starch granules of algae made of branched chains of D-glucose and mannose branches of yeast mannoproteins [34,51]. Therefore, we foresee that accelerated FS could also be employed for the reliable localization of ECM polysaccharides by using fluorophore-conjugated lectins and/or antibodies. Sample preparation improved in this way might prevent the carryover of epitopes in conventional embedding at room temperature. The latter seemingly influenced the immunogold localization of (1→3) (1→4)-β-glucan in conventionally processed *Cetraria islandica* samples [43] and led to controversial interpretation [38].

The regular localization of mitochondria in pockets of chloroplasts of *Trebouxia* sp. photobionts in both *X. parietina* and *C. gyrosa* [52] is ideally preserved through cryopreparation. This is indicated based on the comparison of pocketed mitochondria confined in pockets of a conventionally chemically fixed chloroplast with the corresponding freeze-substituted structures. Mitochondria in pockets of lobed chloroplasts were reported for some plant species previously [53,54]. Yamane et al. suggested that the arrangement of mitochondria in pockets could be related to physiologic stress factors, but it is unclear how maximizing the mitochondria/chloroplast contact relates to functional metabolism. Lichens are exposed to stresses up to extreme levels, both in the short and long term, and they respond in a symbiotic interaction with their habitats [55]. The “pocketing” of mitochondria might be part of a stress scenario in lichens that does not necessarily occur in isolated photobionts. For isolated, cultured *Trebouxia* sp., mitochondria in chloroplast pockets were not reported, regardless of whether the algae were processed at cryo- or ambient temperatures [29,30,31,56]. Cultured *Trebouxia* sp., stressed due to treatment with sodium chloride, did not show mitochondria organization in pockets either [57].

Lichens expose their crystalline metabolites frequently at the surface of their outer cortex and inside the thalli, where they are studied via SEM in combination with freeze-drying [6], the polishing of chemically fixed samples [58], QFDEEM [47] or cryoSEM [20]. Our initial data indicate that accelerate FS can be added as a powerful tool for the elucidation of crystalline metabolites. In cases of Ca-oxalate crystals in the form of weddellite and whewellite, one would expect that they are able to be preserved even through chemical fixation and resin embedding at room temperature, since they resist the organic detergents involved in TEM sample preparation. Such an expectation is supported by numerous observations of oxalate crystals in TEM sections of plant cells and kidney tissues. Surprisingly, *C. gyrosa*, explored as a model of extremotolerant lichens, did not display crystals in conventionally embedded TEM sections, whereas freeze-fractured cryoSEM [20] and Raman spectroscopy [59] pointed to the fact of medullar whewellite accumulation. In agreement with cryoSEM and Raman spectroscopy, our TEM and EDX data of cryoprocessed *C. gyrosa* displayed a wealth of Ca-containing crystals. Therefore, one may wonder how crystals that are known to dissolve very poorly can “disappear” in a conventional TEM preparation. The answer is obvious if one takes into account the extracellular localization of these crystals. If the preparation technique is unable to preserve the delicate ECM of lichens, the crystals are likely to be washed out as a whole. The preservation of the lichen ECM through low-temperature dehydration and fixation prevents the escape of crystals, whereas cryoSEM and Raman spectroscopy avoid any losses by using frozen-hydrated samples and cryo-state sections, respectively. Another aspect, almost as important as retaining Ca-oxalate crystals in the structurally well-preserved ECM of the cryopreserved lichens, is that the interface between both is not disrupted. Consequently, even two-dimensional imaging of sections provides a clue as to the spatial crystal order. This is an advantage that cannot be taken for granted, since intracellular oxalate crystals, while preserved via resin embedding, might break out of the tissue matrix via sectioning, as happened for transgenic, Ca-oxalate-expressing *Arabidopsis* [60].

As indicated through our initial observations, accelerated FS based on cryoimmobilization offers new opportunities for systematic studies of thallus development, including both the transformation of hyphae and photobiont dynamics. With regard to hyphae, our data resemble observations based on QFDEEM, reporting “honeycomb” hyphae and debris containing “acellular struts” instead of “regular hyphae”. According to Goodenough and Roth, regular hyphae undergo transformation in acellular struts that form fluid-filled capillaries [38]. In the past, acellular hyphae structures of *Usnea longissima* and *Ramalina menziesii* were studied in the context of thallus development using transverse TEM sections of conventionally embedded samples [10]. Accelerated FS can be used in future studies of the role of inherent lysis of regular hyphae in the transition into acellular hyphae, since it preserves the structural details of autolysis, as seen in Figure 4f. It might also be used for comparisons of cell wall autolysis in lichenized hyphae with autolysis in separate fungi cultures [61].

With regard to the photobiont ultrastructure, we report major differences in the morphology and ultrastructure of chloroplasts, their thylakoid membranes and pyrenoids. In contrast to conventional sample preparation at room temperature, these observations are based on the instant immobilization of the living state and gentle, continuous, low-temperature dehydration during the course of FS; the chemical fixation at low-temperature is unaffected by sample dependent dynamics, and parameters such as fixative osmolarity and buffer composition.

Here, a note of caution is warranted; the data generated through accelerated FS aim to demonstrate the potential of the method and its admittance in the “toolbox” for studies of lichen research. Neither the status of hydration nor the level of the lichens’ vitality prior to HPF was measured, and the algal photobionts involved were not identified via sequencing. Although *Trebouxia* sp.is the dominant photobiont in the lichen species used in our studies [47,62,63], other microalgae or different clades of the same species could have contributed to the variety of structural patterns of organelles and their sub-structures seen here. Future studies will be required to distinguish species-specific aspects of algal photobiont ultrastructures from those related to biogenesis and cell physiology. Besides the method for ultrastructural investigation established here, such systematic studies will require the accurate determination of experimental main parameters, such as hydration status, light exposure, temperature and the molecular biological identification of the symbiosis partners.

In summary, cryofixation in combination with FS under agitation can be regarded as a practicable and superior technique for the preservation of the ultrastructure of lichens for studies under TEM. It avoids apparent artefacts that are common in samples processed and embedded at ambient temperature. Most importantly, however, it opens up opportunities for studies of photobionts and mycobionts in functional unity with their cryopreserved ECM, inclusively of crystallized secondary metabolites.

## 4. Materials and Methods

### 4.1. Sample Collection

For method development, lichen species of completely different habitats were chosen: fully hydrated *Xanthoria parietina* was used for the establishment of a suitable cryopreparation protocol. Subsequently, the protocol was challenged via application to two extremophile lichens in an undefined state of rehydration, *Circinaria gyrosa* and *Usnea antarctica*. Notably, every lichen species contains one of the most common photobionts, the *Trebouxia* algae [47,62,63].

*X. parietina* sp. (Figure S7a) was collected in March 2019 and April 2022 from the bark of branches of wild shrubs and fruit trees in Deutsch-Wagram and Eggenburg, Lower Austria, and kept in humid conditions up to the time point of high-pressure freezing (hours).

The vagrant lichen species, *C. gyrosa* (Figure S7b) [64], was collected in March 2017 from clay soil in the steppic highlands of Central Spain (Zaorejas, 40°44.691′ N, 02°11.109′ W, 1293 m a.s.l.), which are characterized by extreme insolation, high-temperature contrasts and arid summers [65].

Samples of the lichen *U. antarctica* Du Rietz were collected from the substrate (stones) in January 2017 at James Ross Island, Antarctica (Figure S7c). The selected locality was close to the Czech Antarctic Station of J. G. Mendel (63°48′02″ S, 57°52′57″ W), at an elevation of approximately 10 m a.s.l.

*C. gyrosa* and *U. antarctica* (Figure S7b,c), dried for long-term storage at −20 °C, were rehydrated with deionized water for 60 h at room temperature (RT) with exposure to natural light. Notably, the ability of the photobionts of *C. gyrosa* and *U. antarctica* to recover its photosystem II was studied with a PAM fluorimeter in the past [66,67].

This timespan was considered as sufficient to recover from rehydration, since, after a longer period of rehydration, most phototsymbionts resume photosynthesis after ca. 24 h. For this, the water status in the thalli should be 70% or more.

### 4.2. Chemical Fixation and Embedding at Room Temperature

For comparison with the cryo-preparations, the lichens were processed according to a slightly modified protocol by de los Ríos and Ascaso [4]. Samples from the thalli were fixed with 2.5% glutaraldehyde in 0.1 PBS buffer, pH 7.1, for 3 h, followed by buffer washes and osmification with 1% OsO_4_ in PBS for 2 h. Subsequently, the samples were washed and dehydrated in an ascending series of ethanol. Prior to embedding, pure ethanol was replaced by acetone. For embedding, low-viscosity epoxy resin (Agar Scientific, Stansted, UK) was chosen. The samples were infiltrated as follows: 1 part resin in 2 parts acetone and 1 part resin in 1 part acetone for 1 h each, followed by two parts resin in 1 part acetone overnight. After infiltration with pure resin for 6 h, the samples were polymerized at 65 °C for 36 h.

### 4.3. Freezing

For HPF, cross-sections of the lichens’ thalli were dissected either by hand with razor blades or, for more defined samples, at 250 µm thicknesses with a semi-automatic vibratome, the Leica VT1200 (LEICA Microsystems, Vienna, Austria). They were transferred into type B carriers (LEICA Microsystems, Vienna, Austria) (6 mm in diameter; 300 mm in depth) and covered with the flat surface of another type B carrier. Notably, the carriers were coated with 1-hexadecene (Merck Sharp & Dome Corp., Kenilworth, NJ, USA) prior to use, and the inside volume was filled up with 10% bovine serum albumin (BSA) (PAA Laboratories GmbH, Cölbe, Germany). The carrier sandwich was inserted into the middle plate of a sample cartridge and high-pressure frozen at ca. 2000 bar with the freezer HPM100 (LEICA Microsystems, Austria).

### 4.4. Freeze Substitution under Agitation

FS was performed in an automatic freeze substitution system, the AFS2 (Leica Microsystems, Vienna, Austria), equipped with an agitation module (Helmuth Goldammer, Cryomodultech e.U., Vienna, Austria) as described by Reipert et al. [35]. Carriers containing the HP-frozen samples were placed onto the frozen substitution medium, 1% OsO_4_ (Science Services, Munich, Germany) in anhydrous acetone (VWR International GmbH, Vienna, Austria), in Sarstedt tubes. Without interrupting the cool chain, the Sarstedt tubes were inserted in the tube holders of the agitation module located in the precooled chamber of the AFS2. The rotor for sample agitation was set at medium speed at 15 V. FS took place under agitation at −85 °C. Notably, FS under agitation was performed in the absence of an ethanol bath as a mediator of cold in the cryochamber. Consequently, a significant temperature gradient in the cryochamber had to be considered for the programming of the temperature/time course of the AFS, measured with a K-type thermocouple and USB data logger, EL—USB-TC—LCD (Lascar Electronics, Erie, PA, USA), inside a dummy tube filled with acetone (described in [35]). Fully hydrated *X. parietina* was chosen for testing two temperature/time schedules differing in the duration of FS at ca. −85 °C (Table 1). Since the long-term protocol represented by the corresponding temperature/time curve (Figure S2) proved to be suitable, it was also applied to partially rehydrated *C. gyrosa* and *U. antarctica*.

The infiltration with low-viscosity epoxy resin or Agar100 (both from Agar Scientific, Stansted, UK) was performed step-wise in solvent/resin mixtures, as described above. Notably, the choice of the resin, whether low-viscosity epoxy resin or Agar100, does not influence the quality of infiltration of the samples. Polymerization in freshly prepared, pure resin was accomplished over at least two days in an oven at 65 °C.

### 4.5. Light Microscopy

For the preselection of sample areas, light microscopic sections (2,5 µm in thickness) were cut with a glass knife. Alternatively, thin sections (150 nm in thickness) were cut with a diamond knife. Thick sections were placed on droplets of double-distilled water on glass slides, dried on a heat plate and subsequently stained with toluidine blue for ca. 3–5 s. Alternatively, thin sections floating on water (150 nm in thickness) were taken with a perfect loop (Science Services, Munich, Germany) and placed on glass slides that were warmed up to 70 °C for the rapid evaporation of the water droplet.

Notably, both techniques had their drawbacks caused by surface tensions during section transfer to the glass slide. Thin sections displayed just faint staining in comparison with thick sections, even after the prolongation of the time for staining (see Figure S8). After drying overnight, the sections were covered with coverslips by using droplets of epoxy resin as a mounting medium. Images were taken with a Nikon Eclipse Ni-U and the NIS-Elements image software, version no. 5.11.03 64 bit (Build 1373) (Nikon Corporation, Tokyo, Japan).

### 4.6. Sectioning

The TEM sections were cut with diamond knives. Since compression and local tensions were seen as a major reason for section damage, an ultrasonic oscillating diamond knife, the Diatome Ultra Sonic (Diatome Ltd., Nidau, Switzerland), was applied at a resonance frequency of ca. 26,0 kHz and an amplitude of 6 V. The use of the oscillating knife is described by Studer and Gnaegi [41]. Accordingly, the sample width should be trimmed less than 0.5 mm, which is a limitation in the cross-sectioning of whole thalli. Larger sections might be cut, but they could display damage locally, depending on the lichen species and the degree of hydration. We used the oscillating knife to generate both TEM and LM sections that were 70–100 nm and 150 nm in thickness, respectively. Notably, the latter exceeded the application range recommended by the manufacturer but did not cause knife damage. The TEM sections were placed on copper grids, 200- or 100-mesh wide (Agar Scientific, Stansted, UK), coated with Formvar (Agar Scientific, Stansted, UK), to avoid structures being torn apart during transfer and drying.

### 4.7. Contrasting and TEM

The TEM sections on grids were contrasted with 4% neodymium(III)-acetate [68] for 50 min followed by lead citrate for 8 min, prior to analyses in a TEM EM 900N (ZEISS, Germany) at 80 kV and a TEM Libra 120 (ZEISS, Germany) at 120 kV. Images were acquired using digital cameras, TRS (4 megapixel) and the ImageSp professional software (https://sys-prog.com/en/software-for-science/imagesp/, accessed on 9 August 2023, both Tröndle, Moorenweis, Germany).

## Figures and Tables

**Table 1:** **Two** temperature/time schedules (bold) for FS under agitation tested for cryopreparation of *X. parietina*. Suitability of the long FS protocol was confirmed through application to *U. antarctica* and *C. gyrosa*.

**Short FS under agitation**	**Long FS under agitation**
HPF sample transfer onto liquid N_2_-frozen substitution medium (1% OsO_4_ in acetone) in Sarstedt tubes	
Insertion of the filled tubes in the agitation module within the precooled AFS (ca. −104 °C programmed)	
**FS for 10 h at** **−** **85 °C**	**FS for 60 h at** **−** **85 °C**
Warming up slope (5°−6 °C/h)	
−90 °C for 2 h	
Warming up slope (15 °C/h)	
60 °C for 2 h	
Warming up slope to room temperature (20 °C/h)	
1 h room temperature (to enhance contrast by OsO_4_)	
Removal of the Sarstedt tubes from the AFS for washing and embedding of samples at room temperature	

## Data Availability

The data presented here are available on request from the corresponding author.

## Supplementary Materials

The following supporting information can be downloaded at https://www.mdpi.com/article/10.3390/plants12234039/s1: Figure S1: High-pressure frozen *X. parietina* freeze-substituted under agitation overnight (short protocol); Figure S2: Warm-up curve for FS under agitation in an AFS2; Figure S3: Algal photosymbionts of *U. antarctica* preserved by HPF and accelerated FS; Figure S4: Energy dispersive X-ray microanalysis of cryopreserved lichens embedded in epoxy resin; Figure S5: Lichen preparations by conventional chemical fixation and resin embedding at room temperature; Figure S6: Algal photobionts of *X. parietina* preserved by HPF and accelerated FS; Figure S7: Macrophotographs of the lichens used for FS; Figure S8: Light microscopic resin sections of cryopreserved lichens stained with toluidine blue.

## Abbreviations

AFS—automated freeze substitution system; EM—electron microscopy; EDX—energy dispersive x-ray spectroscopy; FS—freeze substitution; HPF—high-pressure freezing; LM—light microscopy; LTSEM—low-temperature scanning electron microscopy; QFDEEM—quick-freeze deep-etch electron microscopy; TEM—transmission electron microscopy.
