# Supplementary material for: Freeze Substitution Accelerated via Agitation: New Prospects for Ultrastructural Studies of Lichen Symbionts and Their Extracellular Matrix
Source: Plants (Basel). 2023 Nov 30;12(23):4039. doi: 10.3390/plants12234039 (PMC10708280; doi:10.3390/plants12234039)
Supplement: Supplementary file 1 [file plants-12-04039-s001.zip › Supplement Legends.pdf]

Figure S1. High-pressure frozen *X. parietina* freeze-substituted under agitation overnight (short protocol). **a**) overview, displaying most photobionts with small, electron-lucent gaps between the cytoplasm and the cell wall (arrows) and holes in the resin section associated with hyphae (arrow heads). **b**) Algal photobiont accidentally detaching from its cell wall. Arrowheads mark electron- lucent gaps between the cell wall and the cytoplasm. The retracting section of the cell overlaps locally (arrow). **c**) detail of a photobiont displaying numerous small “bridges” between the balk material of the cell wall and the cell membrane (arrows). **d**) Hyphae affected by disruption of their cell membranes from cell walls (marked with arrowheads) and a hole resulting from the loss a complete hypha section profile (asterisk). Scale bars in **a** and **d**, 5 µm, in **b**, 1 µm, in **c**, 500 nm.

Figure S2**.** Warm-up curve for FS under agitation in an AFS2 in the absence of an ethanol bath as a mediator of cold in the cryochamber showing measured temperature changes over time (red line), compared to the programmed temperature/time schedule (blue line), indicating a significant temperature gradient within the cryochamber.

Figure S3*.* Algal photosymbionts of *U. antarctica* preserved by high-pressure freezing and accelerated freeze substitution. Scale bars, 1 µm.

Figure S4. Energy dispersive X-ray microanalysis of cryopreserved lichens embedded in epoxy resin. *C. gyrosa* (**a**,**c**,**e**), *U. antarctica* (**b**,**d**,**f**). **a, d**) Backscattered images. **b,e**) Ca-signal mapping of the areas in **a and b** indicating Ca in *C. gryrosa*, but not in *U. antarctica*. **c, f**) EDX spectra at 20 kV with Ametek (EDAX) detector and APEX Software. a- algae; m- medulla, c- cortex.

Figure S5. Lichen preparations by conventional chemical fixation and resin embedding at room temperature. **a-b**, *X-parietina*; **c-d**, *C. gyrosa*; **e-f**, *U. antarctica*. Scale bars in **a**,**c** and **e**, 5 µm. Scale bars, in **b**, **d** and **f**, 1µm.

Figure S6. Algal photobionts of *X. parietina* preserved by high-pressure freezing and accelerated freeze substitution. Note algal division in b. Scale bars, 1 µm.

Figure S7**.** Macrophotographs of the lichens used for FS: **a**) *X. parietina*, **b**) *C. gyrosa*, and **c**) *U. antarctica.* Scale bars, 1 cm.

Figure S8. Light microscopic resin sections of cryopreserved lichens stained with toluidine blue. **a-c**: *X. parietina*; **d**- *C. gyrosa*; **e** and f: *U. antarctica*. Sections in **a** and **e**, 2,5 µm in thickness, taken with a glass knife. All other sections, 150 nm in thickness, taken with a diamond knife. Note pyrenoids in algal photobionts in **c**, and an alga in division in **d** (marked with arrowheads). c- cortex, m- medulla, cc- central cord. Scale bars: **a**, **b**, **e** and **f**, 100 nm; **c** and **d**, 50 nm.
