# Supplementary figures and images for: Freeze Substitution Accelerated via Agitation: New Prospects for Ultrastructural Studies of Lichen Symbionts and Their Extracellular Matrix
Source: Plants (Basel). 2023 Nov 30;12(23):4039. doi: 10.3390/plants12234039 (PMC10708280; doi:10.3390/plants12234039)

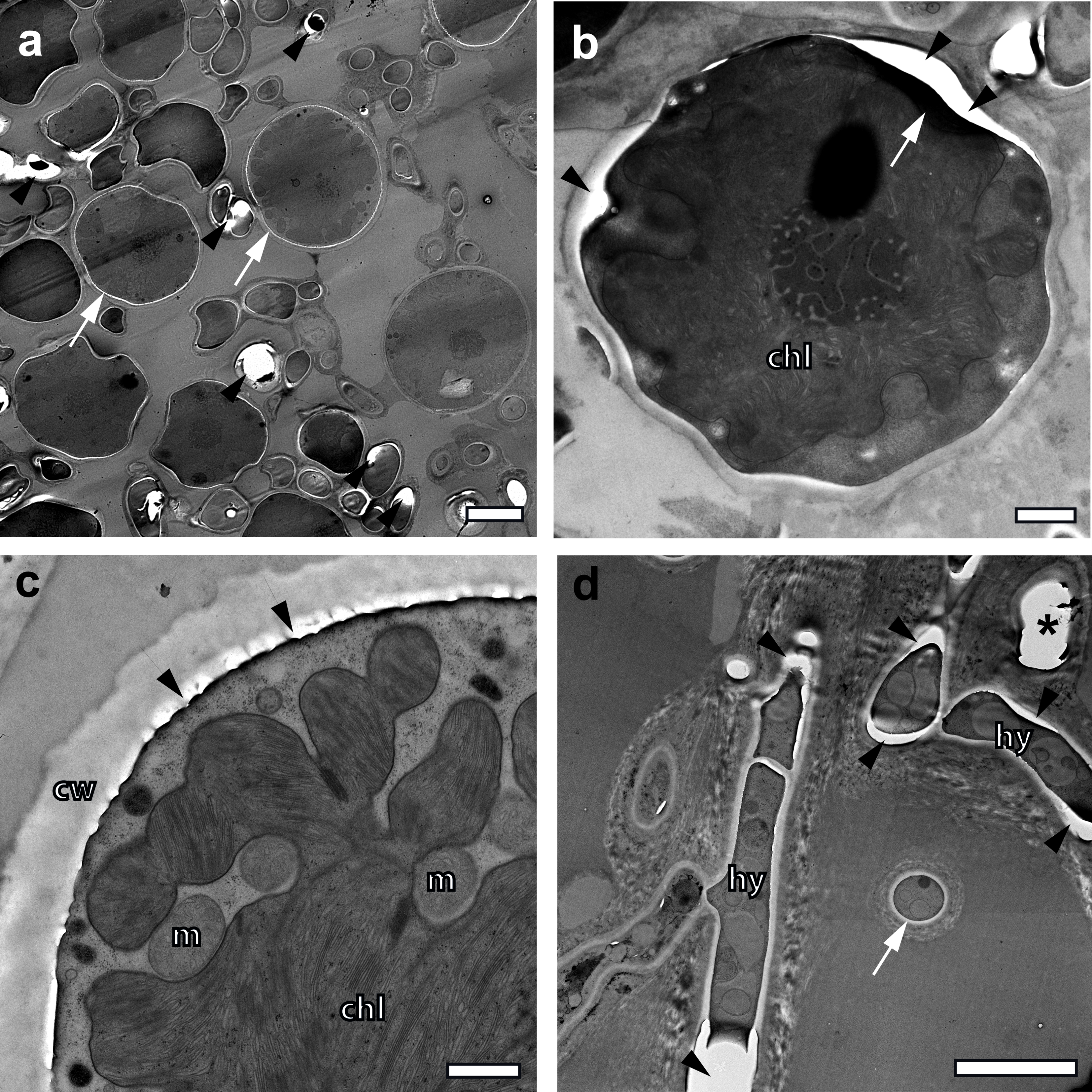

Supplement: Supplementary file 1 [file plants-12-04039-s001.zip › Figure S1.tif]

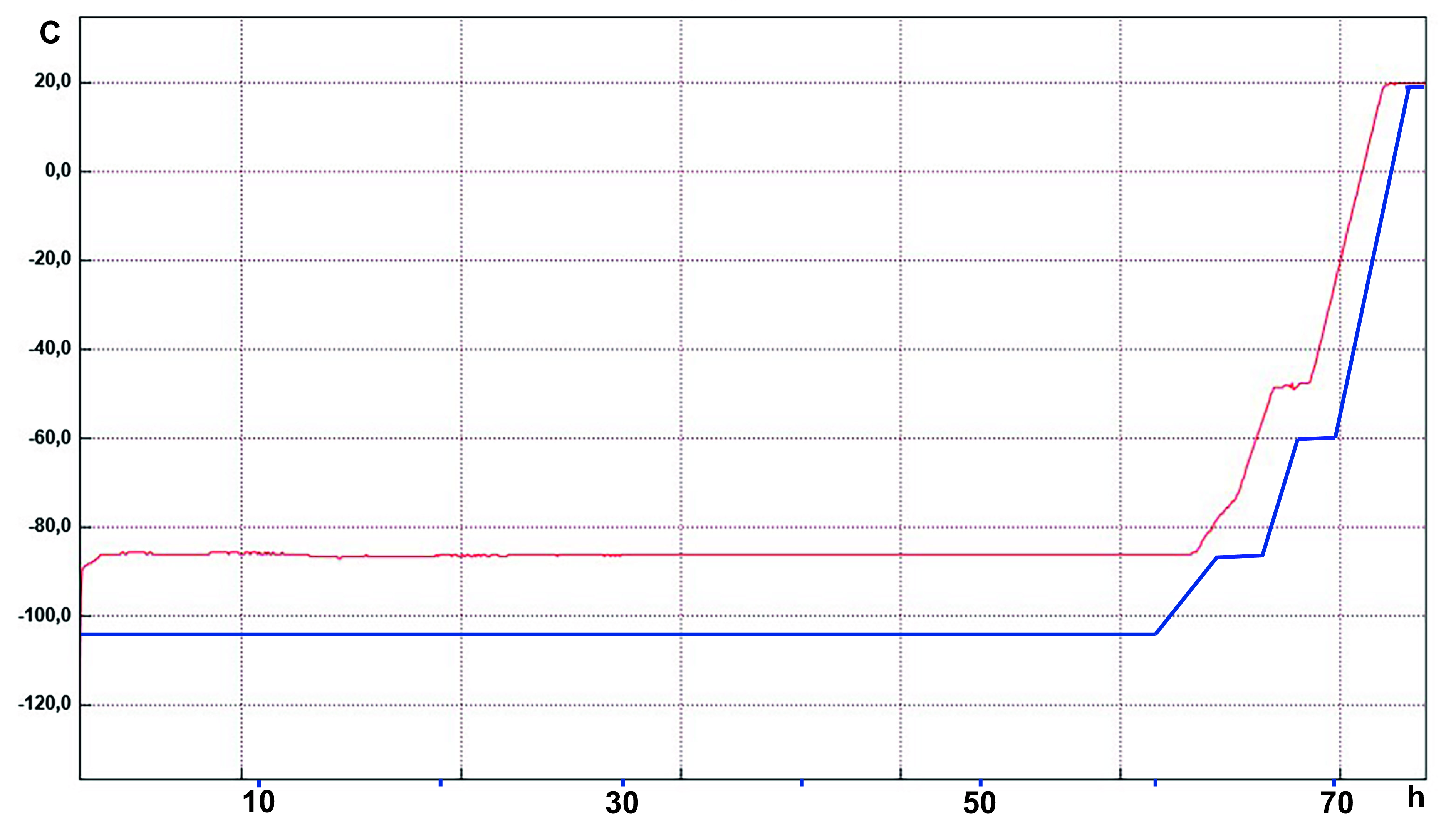

Supplement: Supplementary file 1 [file plants-12-04039-s001.zip › Figure S2.tif]

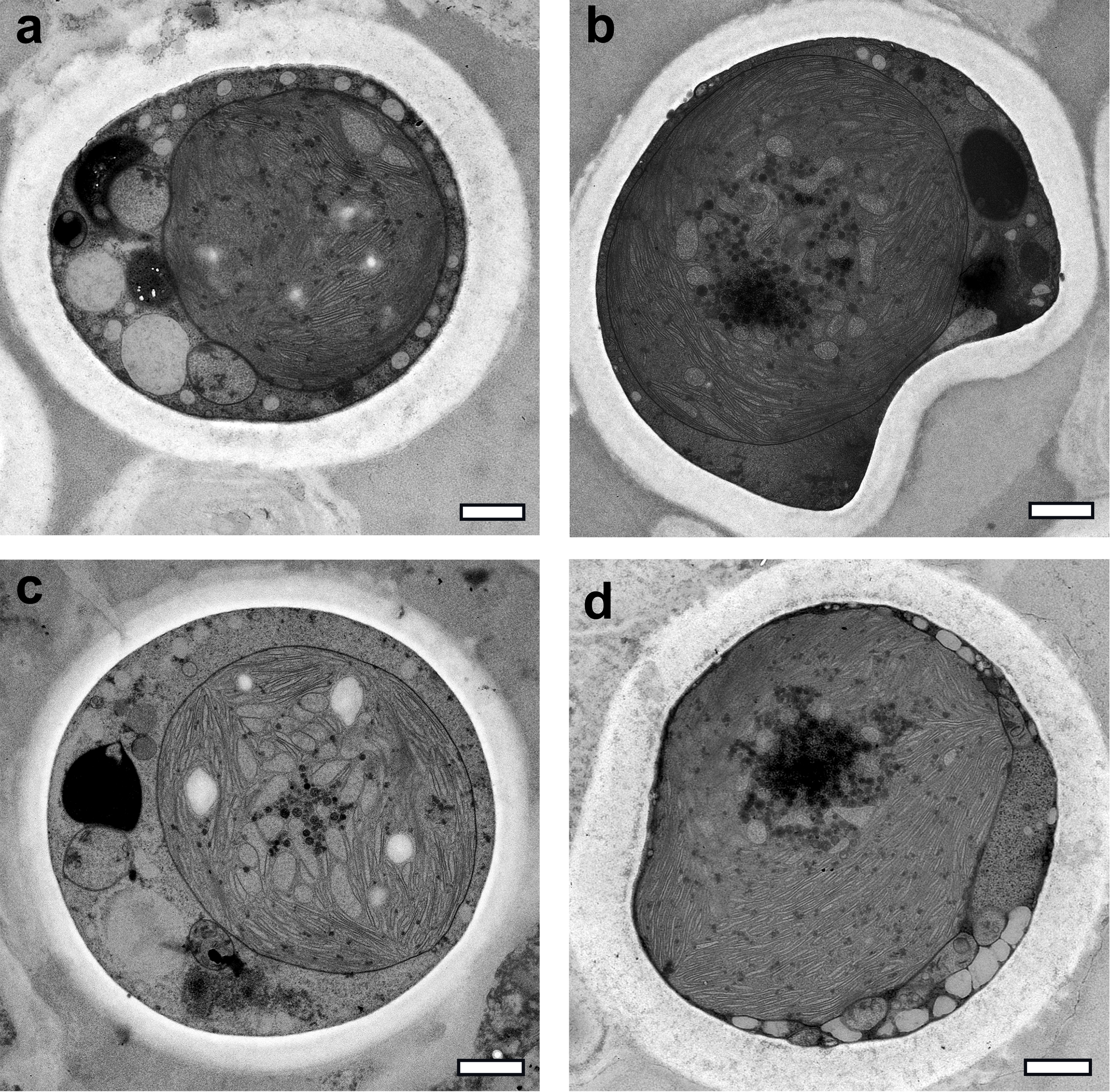

Supplement: Supplementary file 1 [file plants-12-04039-s001.zip › Figure S3.tif]

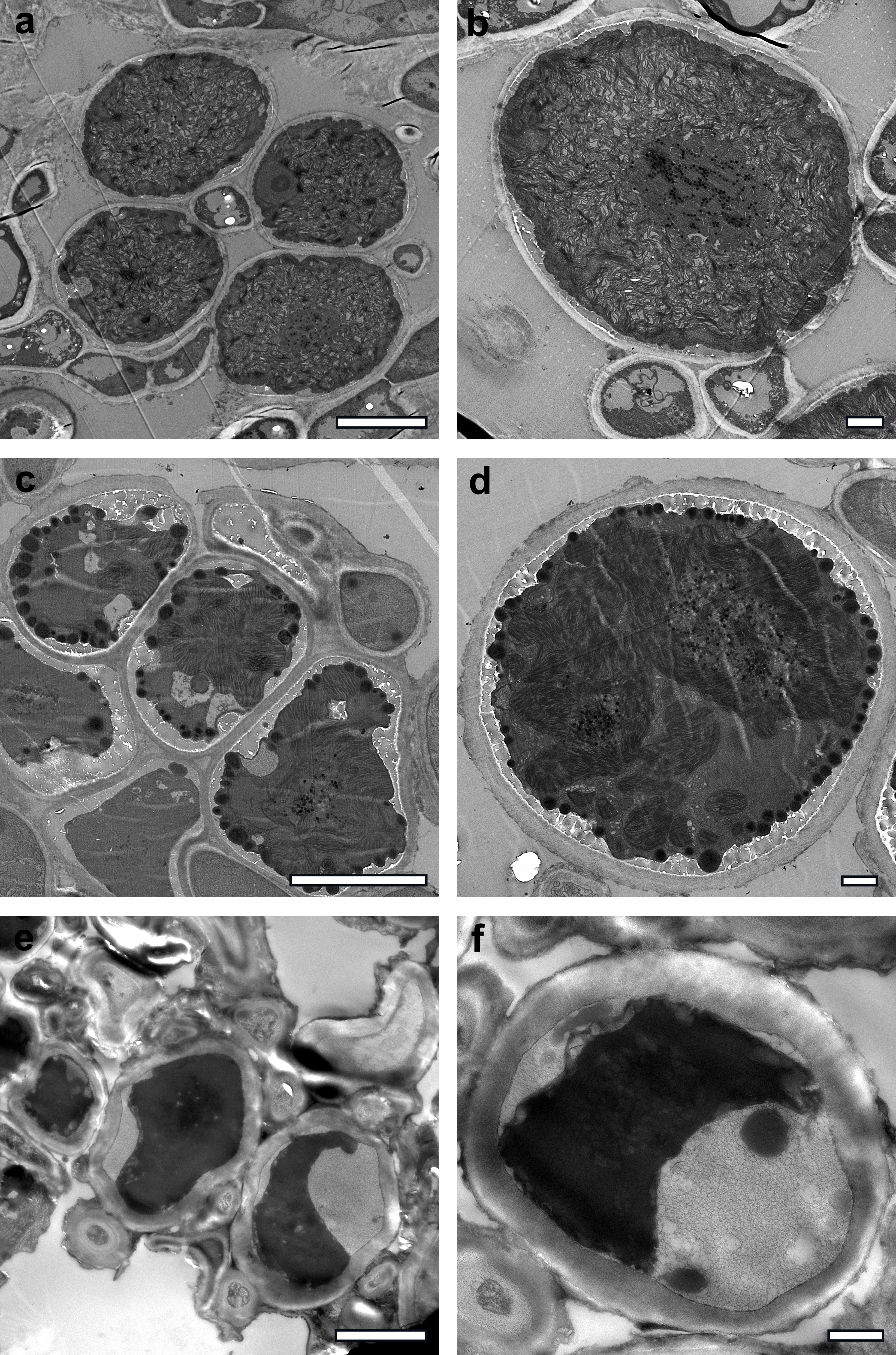

Supplement: Supplementary file 1 [file plants-12-04039-s001.zip › Figure S5.tif]

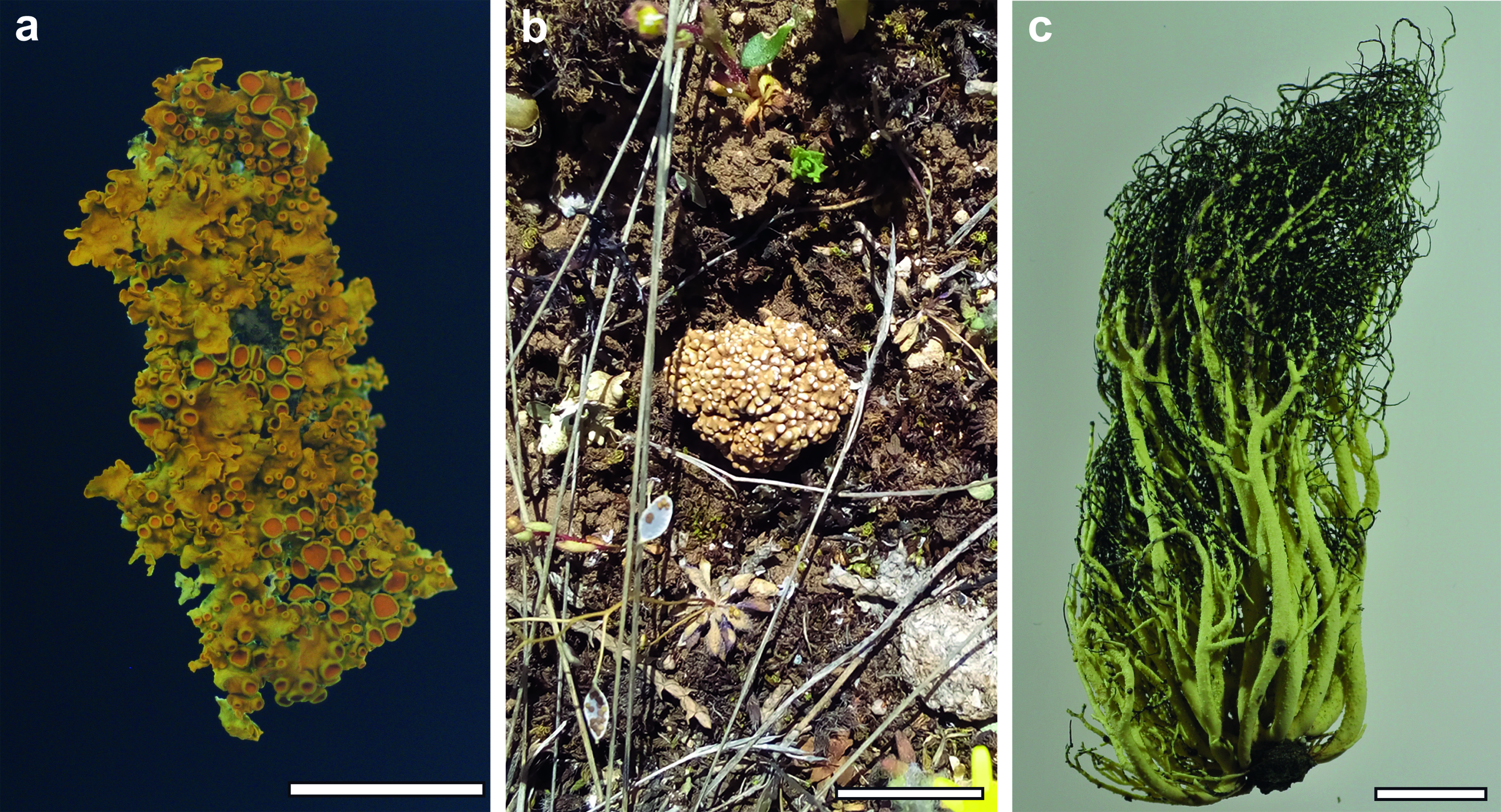

Supplement: Supplementary file 1 [file plants-12-04039-s001.zip › Figure S7.tif]
